# A Facile Design of Colourimetric Polyurethane Nanofibrous Sensor Containing Natural Indicator Dye for Detecting Ammonia Vapour

**DOI:** 10.3390/ma14226949

**Published:** 2021-11-17

**Authors:** Ayben Pakolpakçıl, Zbigniew Draczyński

**Affiliations:** Institute of Materials Science of Textiles and Polymer Composites, Lodz University of Technology, 116 Żeromskiego Street, 90-924 Lodz, Poland; zbigniew.draczynski@p.lodz.pl

**Keywords:** gas detection, natural indicator dyes, protective textiles, sensor, nanofibre, electrospinning

## Abstract

Chemicals and industrial gases endanger both human health and the environment. The inhalation of colourless ammonia gas (NH_3_) can cause organ damage or even death in humans. Colourimetric materials are becoming more popular in the search for smart textiles for both fashion and specific occupational applications. Colourimetric textile sensors based on indicator dyes could be very useful for detecting strong gaseous conditions and monitoring gas leaks. In this study, black carrot extract (BCE) as a natural indicator dye and polyurethane (PU) polymer were used to develop a colourimetric sensor by electrospinning. The properties of the BCE/PU nanofibrous mats were characterized by the Fourier transform infrared spectrum (FTIR) and a scanning electron microscope (SEM). The BCE caused a change in the morphology of the PU nanofibrous mat. To evaluate the colour shift due to NH_3_ vapour, the BCE/PU nanofibrous mats were photographed by a camera, and software was used to obtain the quantitative colour data (CIE L*a*b). The BCE/PU nanofibrous exhibited a remarkable colour change from pink–red to green–blue under NH_3_ vapour conditions with a fast response time (≤30 s). These findings showed that colourimetric nanofibrous textile sensors could be a promising in situ material in protective clothing that changes colour when exposed to harmful gases.

## 1. Introduction

Colour is a powerful communication tool and can be used to signal action. Colourimetric markers have received a great deal of attention in the last decade. They can provide useful information with visible colour changes as a signal in a variety of applications today, from biomedical to safety equipment, where they detect and alert us to changes in the environment [1,2]. According to the Allied Market Research Inc. (Portland, OR, USA) report, the sensor market size was estimated at USD 166.69 billion in 2019 and is expected to reach USD 345.77 billion by 2028 [3]. Colourimetric sensors have attracted the interest of researchers due to their ease of preparation, naked-eye sensing, quick detection, and high sensitivity in response to the growing demand for in-situ analysis [4].

Ammonia (NH_3_) is used in a variety of sectors, including industrial cleaning, chemical production, and food production. Despite its usefulness, it is a hazardous chemical that can cause irritation and burns when inhaled or when it comes into contact with the skin or eyes. NH_3_ gas is difficult to detect with the naked eye because it is colourless and spreads quickly. As a result, in industries that store, handle, or manufacture dangerous substances, continual monitoring is required to identify gas leaks. This situation necessitates study into detecting ammonia gas using a simple approach in everyday situations. Electrochemical, catalytic combustion, semiconductor, and infrared gas sensors are commonly used in numerous industries to detect gas leakage. The demand for portable gas sensors is increasing, and these sensors are being downsized, but their limited portability, issues with lowering power consumption, and high production costs continue to be impediments. Sensors directly applied to textiles are frequently investigated to solve these challenges, with electro-resistive sensors accounting for the majority of these investigations. However, they require a power supply, making them difficult to include in protective clothing [5,6,7,8,9].

It becomes necessary to monitor environmental contaminants accurately, cheaply, and on-site. The use of visual indicators is one of the ways of detecting ammonia gas. The incorporation of these devices as colourimetric gas sensors into textiles is an innovative and intriguing application, and they are part of a new generation of smart textiles for monitoring various volatile chemicals in the environment. Textile materials have numerous advantages, such as flexibility, strength, and lightness, and they can be a good way to develop a colourimetric sensing material for gas leakage monitoring in this context [4,7,8,9].

Detection capability, selectivity, sensitivity, and response time properties are important parameters to produce a colourimetric sensor. Improving the sensing ability of the sensor by increasing its surface area is a way to improve these sensors [10]. The nanofibres have many advanced properties, such as tunable porosity, a high specific surface area to volume ratio, and flexibility of surface functionalization, making them promising candidates for substrates to enhance the performance of the sensors. Electrospinning is a method of producing nanomaterials that use electric force to form continuous fibres with diameters ranging from nanometres to micrometres. In the electrospinning process, a polymer solution is enclosed in a syringe with a linked power source. A high-voltage power source is used to charge the polymer solution. This droplet is drawn into a Taylor cone by the electric field. Polymer droplets break apart and a steady jet forms when the viscosity and surface tension of the solution are suitable [11,12,13,14,15].

Some researchers have focused on the detection performance of the produced sensors in the presence of liquid or gaseous alkalis and acids [16,17,18,19,20,21,22]. Eco-friendly processes and production costs should be considered for commercial textile sensor applications [23,24]. Electrospinning technology has a number of advantages, including ease of use, environmentally friendly processes, and a low cost [25,26,27,28,29,30] for developing textile-based colour-change sensors.

Natural dyes are in high demand for pH-sensitive indicator manufacturing due to their non-toxic and environmentally friendly properties. Anthocyanins are a halochromic class of natural pigments that can generate a variety of colours, including reddish-pink in acidic environments, bluish-purple in neutral mediums, and greenish-yellow in alkaline conditions. Colour changes in these dyes occur because of the phenolic substances—for example, cyanidin, delphinidin, pelargonidin, peonidin, and petunidin—which are exposed to auxiliary changes with a variety in pH [31,32]. Black carrots (*Daucus carota sativus var. atrorubens*) are native to Turkey and the Middle and Far East and have been grown for at least 3000 years. They have been successfully used to colour food industry material due to their high heat, light, and pH stability. In addition, they are plentiful and cheap [33,34,35], which is an advantage in terms of low production costs for the development of atextile-based colourimetric sensor. Several studies have been carried out using anthocyanin extracts from black carrot as a colour-changing nanofibrous sensor. The electrospun polyvinyl alcohol film with anthocyanins from black carrot was developed by Goksen and Ekiz [36]. Moradi et al. [37] investigated the use of an intelligent pH-sensing indicator based on bacterial nanocellulose and black carrot anthocyanins to monitor the freshness of rainbow trout and common carp fillets during storage. Goodarzi et al. [38] developed an intelligent freshness indicator by immobilizing anthocyanins of black carrot within a starch matrix to monitor milk spoilage. Pakolpakçıl et al. [39] blended black carrot extract with polyvinyl alcohol and sodium alginate to develop pH-sensing nanofibrous mats for wound dressing.

**Figure 1:**
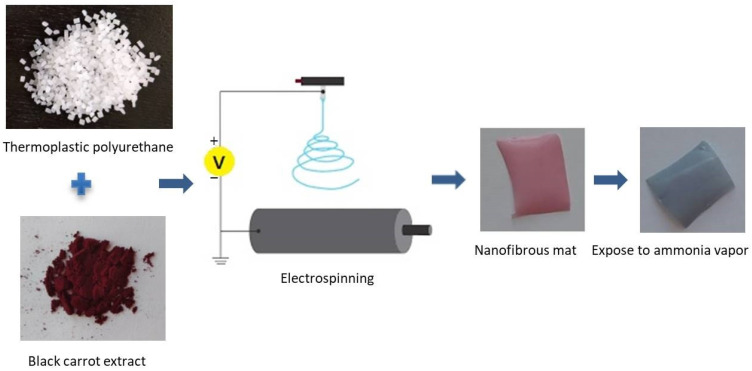
The facile method and experimental study of developing a colourimetric polyurethane nanofibrous sensor containing natural indicator dye for detecting ammonia vapour.

Polyurethane (PU) is an important polymer that is widely used in various fields including automotive, furniture, construction, clothing, and footwear industries. PU is widely used for its low cost, chemical resistance, tear resistance, abrasive resistance, and high load-bearing properties [40,41]. Electrospun PU textiles have been used as filters [42], wound dressings [43], protective clothing [44], packaging [45], and wearable sensors [46]. Developing a colourimetric sensor that utilizes a mixture of black carrot extract and PU might be an option in a gas sensor for protective clothing. There is no previous research based on black carrot extract (BCE) and PU as a colourimetric nanofibrous sensor for NH_3_ vapour in the literature. The electrospinning process was used in this study to produce black carrot extract-loaded PU nanofibrous mats (Figure 1). The electrospun mats’ morphology, chemical composition, and colour change behaviour were investigated. The UV-Vis spectra of BCE solutions in different pH values (pH 2–12) were determined by measuring their absorbance at the maximum wavelength in the visible range. According to the findings, the BCE-loaded PU nanofibrous mat can be considered as an NH3 detector which is critical for human health protection. In practice, the BCE/PU nanofibrous sensor does not require any complicated instruments, electronic components, or even skilled personnel.

## 2. Materials and Methods

### 2.1. Materials

The PU polymer was supplied from BASF (BASF GmbH, Lemförde, Germany) and used in the production of the commercial product (Elastollan^®^ C95, BASF GmbH, Lemförde, Germany) is a polyester-based PU) nanofibrous mat. The BCE was acquired from Pak Natural (Global Inovatif, İstanbul, Turkey). Acetic acid, ammonia (25%), dimethylformamide (98%), sodium acetate trihydrate, sodium dihydrogen phosphate dihydrate, and sodium hydrogen phosphate dihydrate were purchased from Merck (MERCK SP. Z O.O., Warsaw, Poland). All chemicals were used as received, with no further purification. The experimental investigation was conducted with deionized water (DE 20 Plus-Polna, Przemysl, Poland).

### 2.2. Preparation of the Electrospun Nanofibrous Mats

PU polymer was dissolved in dimethylformamide after 24 h of mixing on a magnetic stirrer (Magnetic motor stirrer MS 11, WIGO, Warsaw, Poland) at a concentration of 13% (*w/v*). Following that, two concentrations of black carrot extract (0.25% and 0.5 wt.%) were added to the PU polymer solution. After that, the BCE/PU mixtures were prepared by stirring for 2 h at a 500 rpm stirring rate. The whole solutions preparation process was performed at room temperature ((25 ± 2) °C).

The prepared solutions were placed in a 20 mL syringe with a 0.8 mm inner diameter stainless-steel needle (KDM^®^ KD Medical GmbH, Berlin, Germany). The electrospun mats were produced using a home-made electrospinning machine (Lodz University of Technology, Lodz, Poland). A drum collector covered with aluminium was used to collect electrospun mats. The flow rate and applied voltages were 0.5 mL/h and 35 kV, respectively, during electrospinning, with a distance of 30 cm between the tip of the syringe and the surface of the static collector and a drum speed of 30 rpm. The experimental study was performed in room conditions (relative humidity = (50 ± 5)%; temperature = (25 ± 2) °C). The samples produced were kept in a box at −20°C for further investigations.

### 2.3. Characterization of the Electrospun Nanofibrous Mats

The morphology of the electrospun PU and BCE/PU nanofibrous mats was analyzed by scanning electron microscopy (SEM) (Nova™ NanoSEM 230, FEI Company, Hillsboro, OR, USA). Surface morphologies were imaged at a magnification of 10,000. The diameter distribution and average diameters of the fibres were calculated from the pictures using software (Image J, National Institutes of Health, Madison, WI, USA) with at least 100 measurements per sample.

A Nicolet 6700 spectrometer (Thermo Scientific, Madison, WI, USA) was used to measure the attenuated total reflectance–Fourier transform infrared spectroscopy (ATR-FTIR) of samples. All spectra were recorded within a range of 600–4000 cm^−1^. The OMNIC Specta Software (Thermo Scientific, Madison, WI, USA) was used to further analyse the spectra.

A digital thickness gauge for nonwovens (Checkline J-40-V, Electromatic Equipment Co New York, NY, USA) was used to measure the thickness of the electrospun mats at 5 points. The average and standard deviations of the obtained results were calculated. The test was performed in room conditions (relative humidity = (50 ± 5)%; temperature = (25 ± 2) °C).

### 2.4. UV-Vis Analysis

Before starting the study, the colour-changing property of the purchased black carrot extract was examined. Colour measurements were performed to evaluate the colour properties of BCE at different pH values. For this purpose, 0.1 g BCE was dissolved in 100 mL deionized water, and BCE solution was prepared. Acetate buffers (pH 2–4), phosphate buffers (pH 6–9), and ammonia buffers (pH 10–12) were used to adjust the pH of the medium. Prepared buffer solutions were used in a volume ratio of 1:5 to dilute the BCE solutions. The absorbance of the prepared solutions was measured by UV-Vis spectrophotometer (Lambda 2, Perkin Elmer, MA, USA). In the wavelength range of 400 to 700 nm, absorption spectra were obtained. For the investigation, the SpectraGryph program (v1.2.14, Oberstdorf, Germany) was utilized.

### 2.5. Characterizations of the Colour Change Behaviour of Nanofibrous Mats

The colour change behaviour of BCE-loaded nanofibrous in the presence of ammonia vapour was recorded by using a camera. For this purpose, an approximately 10 mm × 10 mm mat was exposed for 30 s at a distance of around 1 cm above a flask previously filled with 10 mL ammonia solution (25%), and the colour change of BCE-loaded nanofibrous was tracked. Then, the sample was removed from the vapour, and the colour recovered (reversibility) was recorded (Video S1). Photographs were produced from video. They were used for analysis and were taken under the same lighting conditions. The experimental study was performed in room conditions (relative humidity = (50 ± 5)%; temperature = (25 ± 2) °C).

For colour analysis, 50 × 50 pixel samples with the same distance (on the ground) from the image were used. CIE L*a*b* values of the obtained photos were provided by using software (Adobe Photoshop CS5, Phoenix, AR, USA). In CIE L*a*b* colour space values, L* indicates the level of lightness; a* represents the position between red and green, where a positive number indicates red and a negative number indicates green; b* represents the position between yellow and blue, where a b positive number indicates yellow and a b negative number indicates blue. The total colour differences were calculated by the following equation:

Total colour difference (ΔEab*) = [(ΔL*)^2^ + (Δa*)^2^ + (Δb*)^2^]^1/2^ (1)

where ΔEab* is the distance between the two colours in the CIE L*a*b* colour space, and Δ is the difference between the sample’s numeric value and the standard’s numeric value [47].

## 3. Result and Discussion

### 3.1. Characterizations of the Nanofibrous Mats

**Figure 2:**
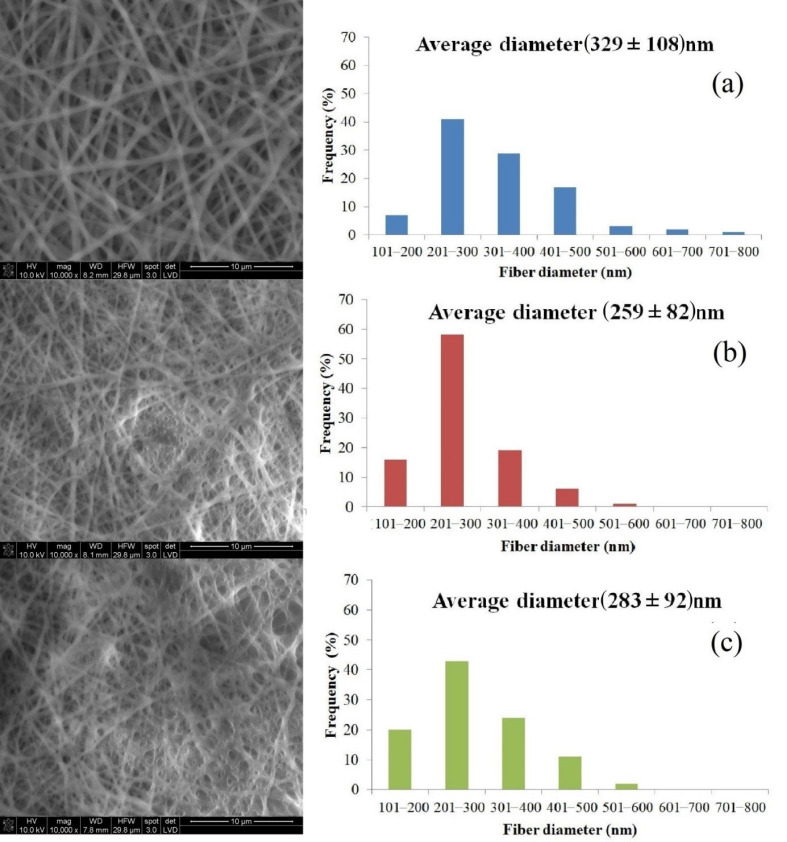
SEM morphology and diameter distribution of the PU and BCE/PU nanofibrous mats with BCE content (**a**) PU (**b**) 0.25-BCE/PU and (**c**) 0.5-BCE/PU.

To evaluate the influence of electrospinning solution composition on the structure of the nanofibrous mat, different concentrations (0.25 and 0.5 wt %) of BCE/PU solution were prepared and used in the fabrication of fibres in this study. The SEM images and the average diameter and fibre diameter distributions of the PU and BCE-loaded PU electrospun mats are given in Figure 2. The structure of electrospun PU nanofibrous was beadless, continuous, and homogeneous (Figure 2a). The average diameter of the electrospun PU nanofibre in this work was 329 nm, which agrees with the findings of Nirmala et al. [48], who reported a similar average diameter for electrospun PU nanofibrous (200–500 nm). When comparing sample SEM images, the presence of BCE in the PU solution had an unfavourable impact on the morphology of mats. The structures of BCE/PU nanofibres were less regular with a few beads, according to SEM pictures of PU containing BCE nanofibres (Figure 2b,c). Agarwal et al. [49] have reported that after adding indicator dyes to Nylon 6 solution, commonly undesired fibre shapes (droplets) formed on electrospun nanoweb surfaces, which could be due to fibre instability or insoluble dyes. The average fibre diameter of BCE-loaded PU nanofibres was found to be in the range of 259–283 nm. BCE includes anthocyanins, carbohydrates, minerals, and vitamins [33]. The presence of ions in solutions may explain the increased conductivity of electrospinning solutions causing the jet to stretch more, resulting in a thinner diameter of nanofibres. Pakolpakçıl et al. [39] have shown that the presence of black carrot extract in polyvinyl alcohol and sodium alginate mixture solution increased the conductivity of the electrospinning solution. In previous studies, at high voltage, solutions pass more quickly through the capillary because the greater electrostatic repulsive forces are exposed, which leads to a defective fibre structure [11,12,13,39,50]. The reason for the reduction in fibre diameter can be attributed to the fact that the BCE/PU solution may have higher conductivity than the PU solution. While the fibre diameter was 259 nm at the 0.25 wt % concentration, the fibre diameter was 283 nm at the 0.5 wt % concentration. As expected, increasing the dye concentration resulted in increased viscosity. The increased viscosity of solutions might be attributed to an increase in solution concentration as well as hydrogen bonding interactions between PU and black carrot extract. Therefore, a change in diameter was observed. While the PU exhibited a variation in the diameters of the nanofibres between 100 and 800 nm, the 0.25-BCE/PU and 0.5-BCE/PU diameters ranged between 100 and 600 nm (Figure 2a–c). The morphologies of PU nanofibres containing different quantities of BCE indicated randomly oriented fibres with a defective structure. Increasing the concentration of BCE had no effect on the morphology of mats. The thicknesses of the PU, 0.25-BCE/PU, and 0.5-BCE/PU electrospun nonwoven fabrics were measured as (64 ± 8) μm, (71 ± 2) μm, and (65 ± 3) μm, respectively.

**Figure 3:**
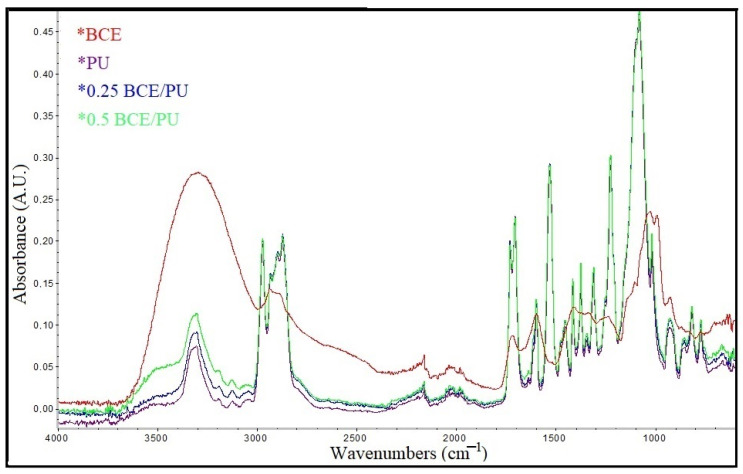
ATR-FTIR spectra of BCE, the electrospun PU, and BCE/PU nanofibrous.

The functional groups of BCE, PU, and BCE/PU nanofibrous mats can be seen in the spectra (Figure 3). The observed peaks in the FTIR spectra of the PU nanofibrous mat were assigned as 3304 cm^−1^ (N–H stretching)_,_ 2970 cm^−1^ (CH_2_ stretching), 1703 cm^−1^ (C=O stretching), and 1222 cm^−1^ (C–O stretching). The peaks are in agreement with the PU reports in the literature [43,48]. The observed peaks in the FTIR spectra of the BCE were assigned as 3300 cm^−1^ (OH groups)_,_ 2937 cm^−1^ (=C–H stretching), 1027 cm^−1^ (C-O stretching), and 923 cm^−1^ (=C–H) [51]. Between the PU and the BCE/PU nanofibres, there were notable differences in peak intensities. With the addition of BCE, the adsorption bands in the frequency range of 3600–3200 cm^−1^ were broadened due to the hydroxyl groups of the groups in BCE. Furthermore, depending on the BCE content, the strength of the absorption band caused by the stretching of –OH groups increased, suggesting that BCE hydroxyl groups were integrated into the PU nanofibrous mats. This result showed an interaction of the molecules in the functionalized nanofibres.

### 3.2. Results of UV-Vis Analysis

**Figure 4:**
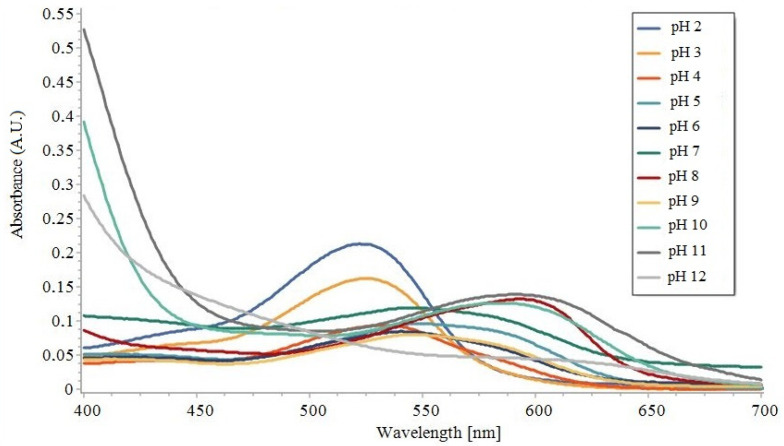
UV-Vis absorbance spectra of the BCE solutions at pH 2–12.

The UV-Vis absorption spectra of the BCE solutions at different pH values are shown in Figure 4. A comparison with the literature indicates that the spectra are typical of anthocyanin pigments, with absorbances in the range of 450–600 nm [52]. The peaks in Figure 4 are in agreement with the UV-Vis absorption spectra of the BCE solutions in the literature [39]. As can be seen, UV absorption was measured at 524 nm and 525 nm, respectively, for pH 2 and 3. The intensity of the peak decreased with increasing pH. At pH 4–6, clear absorbance peaks can be observed at 533 and 550 nm. At pH 7, the absorption band shifted to 553 nm. When the pH was increased to 8, the absorption band shifted to 595 nm along with a broad and increase in intensity. This behaviour was linked to the chemical structural change of black carrots under different pH conditions [37,39]. The maximum absorption peak of the solution was ascribed to flavylium cations at 524 nm (pH 2), whereas the maximum adsorption peak dropped and moved to 553 nm (pH 2–7), which corresponded to the production of quinonoid bases. At pH > 7, second deprotonation produced the carbinol pseudobase, causing the maximum adsorption peak to move to 595 nm. From low to high pH values, a bathochromic change was visible. The colour change behaviour of the BCE solutions was demonstrated using a UV-Vis spectrum, which showed the changes within the medium and could be used to develop a colourimetric sensor.

### 3.3. Colour Change Behaviour of the Electrospun Mats

**Figure 5:**
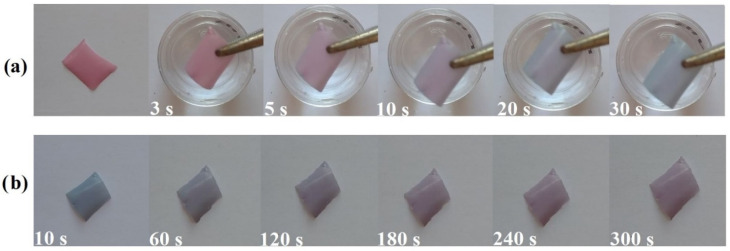
The colour changing performance of BCE-loaded PU nanofibrous mats after exposure (**a**) to ammonia vapour and subsequent removal (**b**).

In industrial operations or personal protective equipment and clothing, the ability to detect alkaline atmospheres quickly with a visual signal is quite useful. To test the NH_3_ sensing capacity, the BCE/PU nanofibrous mat was held in a medium with a certain volume of vigorous liquid ammonia from which NH_3_ may be easily volatilized. The colour change capability was verified and recorded by a camera after the 0.5-BCE/PU nanofibrous mats were exposed to NH_3_ vapour. The video of the colour change performance of the sample is presented in the Supplementary Materials (Video S1). As shown in Figure 5a, the nanofibrous mats were exposed to the NH_3_ vapour. After exposure, the colour of the mats changed. The BCE/PU nanofibrous mats showed a fast response time (≤30 s). The presence of NH_3_ in the environment also provided an alkaline medium on the nanofibre surface. Anthocyanins can change colour depending on the pH of their surroundings. In acidic medium, they usually turn purple–red, while in basic medium, they turn green–blue [53]. The rapid colour conversion with NH_3_ vapour may be related to the large surface area and high porosity of the BCE/PU nanofibrous mats and the fast transformation of black carrot molecular structures. The BCE/PU nanofibrous mats exhibited a good colour change, and colour differences were easily discernible with the naked eye. Furthermore, the colour recovery (reversibility) of the functionalized nanofibres was tested for around 5 min as shown in Figure 5b. The nanofibrous mats became purple within a short time. Due to its different molecular structures, anthocyanin shows colour changes that depend on the variation of pH. At lower pH, the flavylium cation is prominent in anthocyanin structures, contributing to a red colour. Since the pH value is between 4 and 6, the flavylium cation is equalized to the colourless open chalcone form and hydrated to give a colourless carbinol pseudobase. At this pH range, a blend of harmony types of anthocyanin might exist, including chalcone (yellow), carbinol pseudo base (dreary), quinonoidal base (purple or blue), and flavylium cation (red), and this causes anthocyanin to form a purple colour. At pH 6 to 7, the quinonoid bases may deprotonate, and this leads to a bluish quinonoid anhydro base. With further increases in pH, the anthocyanins are corrupted dependent upon their substituent groups. This opens the central pyran ring with dominating anhydro bases, resulting in anthocyanin production of yellow carbinol [32,53,54,55].

The use of CIELab coordinates to determine colourimetric parameters has proven to be a simple and effective method of investigating a textile material’s colourimetric activities. The appearance of a colour in CIELab coordinates provides an objective measurement of the visual perception of that colour. The specification of colour in CIELab values also allows for the evaluation of perceived colour differences or changes [39,47]. Table 1 shows the colour parameters L*, a*, b* with mean values and standard deviations, as well as total colour differences (∆E*).

The BCE/PU nanofibrous mats showed a moderate value of luminosity (L*). The a* colour coordinates of BCE/PU nanofibrous mats showed the highest value at the beginning and then decreased after being exposed to NH_3_ vapour. When exposed to ammonia, the pH value increased. The BCE/PU nanofibrous mats showed negative values of b* value, representing the highest blue intensity. It was noted that 5 min after the sample was withdrawn from the gas source, a* positive value increased and b* negative value decreased, thereby indicating a purple hue. The ∆E* value is important for distinguishing colours at different environmental conditions. Generally, if the ∆E* value is greater than 5, an observer can distinguish between two distinct colours [7,39]. As shown in Table 1, the ∆E* was greater than 5, indicating good detection performance. This finding showed that the produced nanofibrous mats had a remarkable colour variance, allowing the human eye to see colour diversity. Similar colour changes have been seen in other black carrot-loaded nanofibrous materials [37,39]. These findings show that the BCE/PU nanofibrous mats may be used as a textile sensor for detecting NH_3_ gas.

In this work, a naked-eye sensor that does not require a power source or measurement equipment was developed. Therefore, it could have the potential to be used to fabricate a low-cost NH_3_ sensor. Additionally, it can be used to cover large surfaces, and it can still be used as a local sensor with a local colour change without having to include many separate local sensors.

## 4. Conclusions

The goal of this study was to develop a facile colourimetric nanofibrous sensor and evaluate its colour change behaviour to monitor NH_3_ vapour. The results revealed that colourimetric nanofibrous mats were successfully produced by electrospinning with the inclusion of BCE anthocyanin. The presence of BCE caused an irregularity in the nanofibrous mat, which was visible in the SEM images. The average size of the BCE/PU nanofibrous mats was around 250–300 nm. ATR-FTIR spectra of the electrospun mats verified the successful incorporation of BCE in nanofibres. The BCE solution showed colour shifts in the UV-visible absorbance spectra via a bathochromic shift with increasing pH. The BCE/PU nanofibrous had a response to NH_3_ vapour. The visual colour change of the sample with the medium change (ammonia vapour) was remarkable from pink–red to green–blue. The CIELab values of colourimetric nanofibrous mats showed apparent colour differences before and after exposure. This signal may aid workers who are exposed to hazardous conditions in their efforts to protect themselves from gas poisoning. According to the findings, the BCE/PU nanofibrous mat has the potential to be used as a visual indicator for monitoring NH_3_ vapour.

The development of simple, portable, and low-cost colourimetric sensors is a goal of this study for the rapid and in situ detection of environmental pollutants. In this regard, the BCE-loaded PU nanofibrous mat was developed for use as a colourimetric sensor in an unusual environment, and its performance was assessed. Flexible sensors, which could be useful for personal protection equipment, are currently being developed. The primary accomplishment of this study is the development of a technique for preparing colourimetric polyurethane nanofibrous sensors, which will lead to alternative materials for smart textiles. To go further, the stability of anthocyanin-loaded nanofibrous in various mediums (gas concentrations, temperatures, etc.) should be investigated. Understanding the degradation of anthocyanin-loaded nanofibrous under different conditions is critical for the development of a colourimetric sensor.

## Figures and Tables

**Table 1:** CIELab Colour Coordinates and Total Colour Differences of BCE/PU Nanofibrous Mats.

Sample	Colour	L*	a*	b*	ΔE*
Beginning	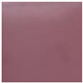	43.8 ± 4.2	22.9 ± 1.7	−1.9 ± 0.6	24.2
Exposed to the NH_3_ Vapour	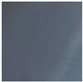	41.1 ± 4.7	−0.1 ± 1.8	−8.9 ± 0.1	-
After recovered	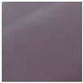	40.3 ± 2.7	8.9 ± 0.1	−4.4 ± 1.0	10.1

The mean values and standard deviations (±) of the three measurements were used. The sample exposed to the NH_3_ gas took as a reference to calculate ΔE* values.

## Data Availability

Not applicable.

## Supplementary Materials

The following are available online at https://www.mdpi.com/article/10.3390/ma14226949/s1, Video S1: The colour changing performance of BCE-loaded PU nanofibrous mats after exposure to ammonia vapour and subsequent removal from the vapour source.
